# Subjective Outcome Evaluation of Instructional Videos in Leadership Education

**DOI:** 10.3390/ijerph20010367

**Published:** 2022-12-26

**Authors:** Xiang Li, Daniel T. L. Shek, Tingyin Wong, Lu Yu

**Affiliations:** Department of Applied Social Sciences, The Hong Kong Polytechnic University, Hung Hom, Hong Kong, China

**Keywords:** subjective outcome evaluation, instructional videos, leadership education, higher education, online learning

## Abstract

The aims of this study were to examine the psychometric properties of two subjective outcome evaluation tools measuring students’ perceptions of 24 instructional videos and to understand the profiles of students’ perceptions of the videos. Online teaching and learning played an important role when school lockdown measures were imposed during the COVID-19 pandemic. To facilitate online teaching in a college-level leadership education subject, we developed and piloted 24 instructional videos, including 15 animated videos and 9 case-based videos, in the 2021/22 academic year. To understand students’ perceptions of the videos, we developed two subjective outcome evaluation scales (one for the animated videos and another for the case-based videos) to assess the subjective perceptions of 1308 students. Results showed that the developed tools possessed good psychometric properties, including factorial, convergent and discriminant validity. The findings of this study also revealed the students had positive attitudes towards the developed videos, including positive perceptions of the videos’ design and the benefits gained from watching them. The present study suggests teachers can meaningfully use the 24 instructional videos in the context of leadership education in higher education.

## 1. Introduction

Hong Kong university students are facing various challenges and encountering different developmental issues, such as personal problems, unhealthy lifestyles, mental health issues and social connection problems [1,2]. Moreover, mental health problems are prevalent among Hong Kong youths, including depression, anxiety and excessive stress [3,4,5], internet addiction [6,7], materialistic beliefs and egocentric behaviors [8]. Unfortunately, efforts to promote life skills and social-emotional learning skills amongst young people are weak in different education sectors, including higher education [9].

With globalization, the changing economic ecology requires students to acquire skills specific to the 21st century [10]. Cornali [11] argued that both hard skills (e.g., job- and discipline-specific skills, technical and cognitive abilities) and soft skills (e.g., creativity, flexibility, resilience, communication and problem-solving skills) are essential to one’s employability. In Hong Kong, the service economy requires employees to have strong intrapersonal and interpersonal competences [12]. However, during the COVID-19 pandemic, the cultivation of soft skills such as interpersonal competence, effective communication and problem-solving skills was particularly difficult because of social distancing and school lockdown measures [13].

At one university in Hong Kong, we have developed a leadership subject entitled “Tomorrow’s Leaders” based on the positive youth development (PYD) approach, which focuses on young people’s strengths and potentials. The PYD approach was conceptualized by Lerner et al. [14] as a developmental process resulting from an individual’s engagement with his/her ecology in a positive manner. According to this view, positive development in young people is shown by five qualities—character, competence, confidence, connection and caring. Moreover, Catalano et al. [15] proposed 15 PYD constructs (e.g., competence in emotional management and social relationships, morality, spirituality and prosocial norms) as basic indicators of PYD. Based on these PYD theories, we developed and launched the 3-credit “Tomorrow’s Leaders” subject in the 2012/13 academic year. This subject consists of 13 lectures covering ten intrapersonal and interpersonal qualities, such as social competence, cognitive competence, resilience, morality, spirituality, self-leadership and law-abidance leadership [16,17]. In each lecture, we use experiential learning pedagogies, including class and group discussion, self-reflection, role-playing activities and case studies, to enrich students’ learning experience. Since the launch of the “Tomorrow’s Leaders” subject in the 2012/13 academic year, we have conducted regular evaluations. For quantitative evaluation, objective outcome evaluations [17,18], subjective outcome evaluations [19] and process evaluations [20] have been implemented. For qualitative evaluation, we have collected data based on personal reflections [21] and focus group interviews [22]. These evaluation studies revealed the effectiveness of this subject in promoting PYD attributes in university students and strengthening their mental well-being.

### 1.1. The Need for Virtual Teaching and Learning Materials

Electronic learning (e-learning) is conceptualized as the use of technology (e.g., media and digital electronic tools) to facilitate learning [23]. Empirical studies show that various technologies (e.g., mobile phone, online platform and 3-D virtual world) have brought benefits to students’ learning [24,25,26]. During the COVID-19 pandemic, school lockdowns negatively affected the learning of nearly 1.6 billion students, or 94% of learners, globally [27]. Therefore, online teaching and learning plays an important role. Online teaching offers synchronous (e.g., the delivery of lectures through video conferencing platforms) and asynchronous (e.g., pre-recorded lecture videos) learning environments [28]. In Beijing Normal University, out of the 4036 courses offered in the semester starting in February 2020, 3238 were delivered online [29]. There are studies suggesting that many applications of online courses during the pandemic were crisis-response migration methods adopted by universities [30,31]. Many teachers and students were not well-prepared for online learning. This situation was particularly severe in developing countries where students faced various technical and monetary issues in online learning [32]. Obviously, there is a need to reflect on the issues and policies that surrounded teaching and learning in an online setting during the COVID-19 pandemic [33,34].

There is a growing trend of video-based learning, where educational videos are combined with different teaching and learning activities [35], such as hands-on skills exercises and virtual case discussions in dental schools [36] and PowerPoint slides, readings and face-to-face practical tutorials in data science labs [37]. Before the COVID-19 pandemic, Kay [38] found that learners had positive affective and cognitive attitudes towards video podcasts (including audiographs, video streaming and webcasting). Scagnoli et al. [39] reported that video lectures enabled students to have control over the media and feel the presence of the instructors, thus increasing their sense of engagement with the online course content. Other studies also supported the effectiveness of video lectures in promoting students’ learning outcomes [40]. During the pandemic, Wikandari et al. [41] found that using animated videos in flipped learning significantly enhanced students’ ability to comprehend and apply biochemistry knowledge. Wu et al. [42] also showed that theoretical knowledge and student satisfaction in the experimental group (video-based program plus flipped classroom) were higher than that of students in the control group. In addition, other studies demonstrated the effective use of instructional videos in reinforcing teamwork activities amid the pandemic [43]. To conclude, although virtual teaching and learning had already shown to offer promising learning outcomes before the pandemic, its teaching and learning effectiveness during the pandemic has not been clearly established. Therefore, the development and assessment of educational videos in a college-level leadership subject during the pandemic could fill this knowledge gap in the literature.

### 1.2. Research Gaps in the Literature

There are three research gaps regarding the use of videos in leadership education, particularly during the pandemic. First, there are very few attempts to develop video-based teaching and learning materials. Jenkins [44] suggested that class discussion, individual projects, group presentations, self-reflections and personal assessments were the dominant pedagogies in U.S. undergraduate leadership education. Within the classroom setting, videos are especially useful for experiential learning [45]. Evaluating the use of video simulation of clinical scenarios (e.g., moral dilemmas, patient management) in nursing education, Sharpnack et al. [46] found that video simulation was able to enhance students’ critical thinking skills and develop other competencies related to leadership and safety. These findings highlight the significant benefits of video-based teaching for university leadership education.

Second, there are relatively few attempts to develop video-based teaching and learning materials for undergraduate programs in Hong Kong. Searching the Web of Science database with the keywords “video-based teaching and learning” and “Hong Kong” on 15 November 2022, there were only 28 citations. Furthermore, most of the papers were on teacher professional training and secondary school students, with only three studies investigating video-based teaching in undergraduate courses [47,48,49]. Moreover, although traditional Chinese students expected didactic teaching and learning, the younger generation increasingly favor interactive pedagogies in recent years. With the use of smart phones and exposure to social media, young people are more receptive to audio-visual teaching materials.

Third, there are very few existing evaluation studies on video-based materials in virtual teaching and learning. Evaluation is needed to ensure the instructional effectiveness of newly developed teaching materials. Howe [50] used a 12-item student survey to assess students’ perceived educational benefits of a CD-ROM with images and instructional videos on basic veterinary surgical skills. However, the psychometric properties of the assessment tool are not clear. Schooley et al. [51] highlighted the lack of standardized scales to measure educational videos’ quality and developed the instructional video quality checklist (IVQC) to assess how multimedia principles have improved students’ learning efficiency. However, the psychometric properties of the scale, particularly its dimensionality, are not clear.

The development of virtual teaching and learning (VTL) materials such as videos was urgently needed during the pandemic due to the heavy reliance on online teaching and learning. With the support of the University Grants Committee, our project team started developing animated videos and case-based videos to facilitate the teaching of “Tomorrow’s Leaders” in 2021. In Semester 2 of the 2021/22 academic year, 15 animated videos and 9 case-based videos were developed and piloted. We have developed more animated videos than case-based videos (15 versus 9) for two reasons. First, since we already had some case videos but no animated video, we developed more animated videos to fill this gap in our teaching materials. Second, studies found that animated videos are appealing to young people, and they can produce significant learning outcomes [52,53,54]. As such, we believed that developing more animated videos could bring significant benefits to students’ learning.

Moreover, because of social distancing measures, this subject was conducted using an online synchronous teaching mode. At the same time, we applied the flipped classroom approach to deliver the 24 videos. Students were required to watch these videos to learn the course content and finish the reflection activities before attending the online lectures. The intention in developing this e-learning module was to enrich students’ understanding of related topics before joining the 2-h face-to-face or online synchronous lectures. A brief description of these videos and reflection activities are listed in Table 1 and Table 2. Each lecture contains at least one animated video and one case-based video (except for the lectures on moral competence and law-abiding leadership which contain no case-based video).

### 1.3. The Present Study

As mentioned above, we have conducted evaluations to measure the teaching effectiveness of the “Tomorrow’s Leaders” subject since the 2012/13 academic year. One evaluation strategy is the application of a subjective outcome evaluation to measure students’ satisfaction with and perceptions of the instructional effectiveness of the subject via the client satisfaction approach [19]. Researchers in many fields (including education and social work) have applied the client satisfaction approach to assess the degree to which consumers are satisfied with the process and outcomes of an intervention program. It is also used to measure service recipients’ perceptions of how their needs are met after an intervention [55]. In higher education, studies have emphasized the importance of assessing education quality from the students’ perspective. Wang, Sun, & Jiang [56] argued that students’ perceptions of education quality could be divided into three dimensions—students’ engagement in learning, students’ satisfaction and students’ evaluation of teaching. A previous study revealed that subjective outcome evaluation was significantly associated with objective outcome evaluation [57]. Zhu and Shek [58] also highlighted several advantages of subjective outcome evaluation, such as efficiency, cost-effectiveness and significance through data accumulation [58].

In this study, based on an integration of the previous studies and existing measures, we developed two subjective outcome evaluation scales to assess students’ perceptions of the 24 videos: one for the animated videos (12 items) and one for the case-based videos (14 items). Each scale measures the content, implementation quality and effectiveness of the videos. Since the 24 videos were part of the lectures, two items were added to evaluate students’ perceived effectiveness of each lecture. There are two research objectives of this study:

1. To examine the psychometric properties of two subjective outcome evaluation scales for evaluating animated videos and case-based videos.
2. To evaluate students’ perceived quality of 24 newly developed videos in terms of their content, implementation and effectiveness using the two subjective outcome evaluation scales.

## 2. Materials and Methods

To facilitate online teaching of the “Tomorrow’s Leaders” subject, our project team developed and piloted 15 animated videos and 9 case-based videos. These videos were uploaded to a digital teaching support system, “Blackboard Learn”, along with other e-learning modules of the subject. Moreover, two evaluation scales, including one for animated videos and another for case-based videos, were designed and uploaded to the e-learning platform. Students were invited to complete the evaluation forms after watching the videos every week in a voluntary and anonymous manner. The psychometric properties of these scales and students’ perceptions of the videos were analyzed at the end of the semester.

### 2.1. Participants

In the second semester of the 2021/22 academic year, 1308 Year 1 undergraduate students took “Tomorrow’s Leaders.” These students studied at the following seven schools or faculties: School of Fashion and Textiles, School of Design, School of Hotel and Tourism Management, Faculty of Science, Faculty of Health and Social Sciences, Faculty of Engineering and Faculty of Construction and Environment. To maintain anonymity of the participants and to encourage their participation in the evaluation, we did not collect demographic information from the students. Generally speaking, Year 1 students of the university have a mean age of 18 and a gender ratio of roughly one female to one male. For each of the 24 videos, 598 to 1114 students evaluated the video. For 20 of the videos, at least 900 students provided their evaluation. Table 1 and Table 2 show the content of the videos.

### 2.2. Procedures

We obtained ethical approval from the institutional review board of the university and consent from each participant before data collection. “Tomorrow’s Leaders” consists of 13 lectures, including one lecture for introduction, 10 lectures for different topics related to leadership and two lectures for student group presentations. From Lecture 2 to Lecture 11, all students were required to watch two to three animated or case-based videos in the e-learning module before attending the lecture each week to fulfil the learning requirement. Meanwhile, we invited all students to complete the two evaluation scales plus two items on the lecture’s effectiveness in a self-administration manner.

### 2.3. Instrument

We created two evaluation scales, including one for animated videos and another for case-based videos. We designed these measures with reference to the Subjective Outcome Evaluation Scale (SOES) specifically developed for participants to evaluate the quality of a project in terms of its content, implementation and effectiveness [19,57,58]. While we used 12 items for the animated videos (e.g., “the animated video has helped me to reflect on the topic”), we used 14 items to evaluate the case-based videos (e.g., “the video-based activity has enabled me to apply the theories in real life cases”). There are two more items for case-based videos because case-based videos contain classroom discussion during the lectures. As such, items assessing the interaction between the teacher and the students and the interaction between the students were included in the scale for case-based videos. Moreover, two items were added to measure the effectiveness of each lecture. For each item, the students rated it on a six-point Likert scale, with 1 representing ‘strongly disagree’ and 6 representing ‘strongly agree’. Higher ratings indicate a better appraisal of the quality of the videos by students. The reliabilities of the two scales were excellent, with scores ranging from 0.96 to 0.99 for the 15 animated videos and 0.97 to 0.99 for the 9 case-based videos.

### 2.4. Data Analytic Plan

In this study, Mplus Version 8.8 was used to conduct confirmatory factor analyses (CFAs) to assess the two rating scales’ factorial validity. To evaluate the one-factor model for each model, we examined a series of model fit indices, including the comparative fit index (CFI), the non-normed fit index (NNFI), the standardized root mean square residual (SRMR) and the root mean square error of approximation (RMSEA). For CFI and NNFI, equal or above 0.90 values indicate a good fit; for SRMR and RMSEA, equal or below 0.08 values imply a close fit [59].

Furthermore, convergent and discriminant validity were assessed by calculating the average variance extracted (AVE). AVE ≥ 0.50 represents a favorable convergent validity. Reliability of the scale was evaluated by calculating the composite reliability (CR). CR ≥ 0.70 indicates a good reliability [60,61]. We performed separate factor analyses for the responses to each of the 24 videos.

After confirming the validity and reliability of the two rating scales, we used the two scales and the two items on lecture effectiveness to evaluate students’ perceptions of the content, implementation and educational effectiveness of the 24 videos. Descriptive statistics, particularly percentages of positive responses to each item of the scales, were conducted for each video. Our previous studies on subjective outcome evaluation adopted a similar approach to measure students’ perceptions of the quality and effectiveness of the “Tomorrow’s Leaders” subject [17,19].

## 3. Results

### 3.1. Psychometric Properties of the Two Evaluation Tools

The findings of the CFAs showed that the one-factor model fits the data very well for both scales of the animated videos and case-based videos separately (see Table 3 and Table 4). For the 15 animated videos, the one-factor model of the 12-item measure showed a good model fit: CFIs ≥ 0.94, NNFIs ≥ 0.92, SRMRs ≤ 0.04 and RMSEAs ≤ 0.08. Good AVEs ≥ 0.65 and CRs ≥ 0.96 were found. For the nine case-based videos, the one-factor model of the 14-item measure showed a good model fit as well: CFIs ≥ 0.93, NNFIs ≥ 0.92, SRMRs ≤ 0.04 and RMSEAs ≤ 0.07. Good AVEs ≥ 0.66 and CRs ≥ 0.96 were also found. All model fit indices supported the one-factor model of the scales for both animated videos and case-based videos.

### 3.2. Client Satisfaction Responses

For the animated videos, the findings revealed most of the students had positive views towards the content of the videos and their implementation and educational effectiveness (Table 5). For example, for the item “the animated video is interesting”, the mean scores of the 15 animated videos ranged from 4.53 to 4.9, and the positive response rates varied between 90.81% and 96.65%. In addition, for the item “the animated video can benefit my development”, the 15 videos received mean scores ranging from 4.68 to 4.95, with positive response rates varying between 93.58% and 97.94%. These findings suggested that the majority of students had positive perceptions of the animated videos’ content, the delivery of the videos in the online modules and the contribution of these videos to their learning in this subject.

Similarly, for the case-based videos, students had a good appraisal of the video content, implementation and their educational effectiveness (Table 6). For example, for the item “the content of the video is stimulating”, the nine case-based videos received mean scores from 4.70 to 5.01, with positive response rates varying between 96.05% and 98.02%. In addition, the item “the video-based activity has enabled me to apply the theories in real life cases” had mean scores ranging from 4.68 to 4.99, and its positive response rates varied between 93.60% and 97.67% across the nine videos. These figures revealed the majority of students had positive views towards the content of the case-based videos, their utilization as well as educational effectiveness.

Finally, the last two items measured students’ perceptions of the general effectiveness of the lectures. These two items received highly positive responses (Table 7). For example, the topic of Lecture 3 is “Cognitive Competence”, and it contains two videos, “Cognitive Competence” and “Myths of MMR Vaccine”. The findings showed that 97.44% of students perceived the lecture had enriched their development, with a mean score of 4.79. The evaluation results showed that students were very satisfied with the integration of the 24 videos in “Tomorrow’s Leaders” lectures.

## 4. Discussion

To enrich the teaching materials for a leadership subject, we developed 24 instructional videos (15 animated videos and 9 case-based videos) and conducted an evaluation of the developed videos. There are a number of special features of this study. First, as there are few instructional videos for leadership education in Hong Kong universities and colleges, we filled this gap by developing videos with reference to different aspects of leadership qualities based on the positive youth development perspective. Second, to promote the learning motivation of students, we developed both animated and case-based videos. Third, we conducted a subjective outcome evaluation of the videos based on a large-scale student survey. Fourth, we conducted confirmatory factor analyses to support the psychometric properties of the two measures for the animated videos and case-based videos. Fifth, the evaluation findings provide support for the implementation of instructional videos in teaching leadership in the higher education sector.

Conceptually, the evaluation results from this study support the proposition that instructional videos are useful in teaching and learning. In the literature on instructional videos, there are views suggesting the positive value of instructional videos in promoting student motivation and teaching effectiveness [36,37,38,39,40,41,42,43]. During the pandemic, Doudesis and Manataki [37] found that undergraduate students preferred video-based online labs over instructor-led online labs in data science. Students enjoyed video-based online labs more and found them to be more useful. Wu et al. [42] revealed that relative to traditional teaching methods, college students in a video-based plus flipped classroom program showed significantly higher scores of theoretical knowledge, overall satisfaction, learning efficiency and enthusiasm, as well as problem-solving and independent learning abilities. The present study supports the existing literature on the benefits of instructional videos on students’ learning. Furthermore, the present findings expand the scope of the value of instructional videos to university leadership education in a Chinese cultural context. Of course, there is a need to understand the mechanisms involved in generating the positive impact of instructional videos, such as increasing students’ control over learning and improving their study habits [39]. Moreover, this study further supports the importance of enriching online teaching and learning for students during the pandemic. The study by Leung, Shek and Dou [62] illustrated the delivery of service-learning in an online setting during the pandemic, and the subjective and objective outcome evaluation in the study found that both college students and service recipients benefited from the online service-learning. The current study also demonstrated that instructional videos conducted in an online setting could benefit college students during this unprecedented time.

Methodologically, the present study generated two measures of subjective outcome evaluation—one for animated videos and one for case-based videos. As there are few validated tools for the evaluation of instructional videos, the findings of this study are innovative additions to the existing literature. Particularly, studies that examine related evaluation tools are almost non-existent in the current scientific literature. For example, Howe [50] and Schooley et al. [51] developed scales to measure students’ perceptions of instructional videos’ quality and their learning efficiency using these videos. However, the psychometric properties and the dimensionality of their assessment tools are unclear. There are three reasons why we should have validated assessment tools for instructional videos. First, we need scientific tools to measure students’ perceptions. By listening to student responses, teachers can have more dialogues with students on the subject matter that would further promote the teacher–student relationship. Second, it is common for tertiary education institutions to conduct subjective outcome evaluations, which is also regarded as a routine mechanism for quality assurance. Although studies have argued that the concept of quality in higher education is very complicated, it is still apparent that students are the most important stakeholders [63]. Moreover, similar subjective outcome evaluation tools have been widely used [17,19,64]. Third, evaluation findings provide cues on how teachers could improve the instructional materials as well as their teaching. It is noteworthy that some teachers used subjective outcome evaluation to evaluate the hybrid mode of teaching [65] and service-learning [66] in connection with leadership education.

Practically, the present study demonstrates the possibility of developing animated and case-based videos for leadership education built from the positive youth development theories. Because positive youth development attributes significantly contribute to the healthy development of young people and form the foundation of effective leadership, nurturing students’ positive youth development attributes is important [16]. The present study suggests the value of using instructional videos to impart knowledge on PYD. In addition, as young people welcome images (e.g., Instagram), using educational videos can facilitate college students’ learning. In particular, research has suggested the significant educational effectiveness of using instructional videos during the COVID-19 pandemic, which is still affecting many people around the world [41,42,43].

Despite the pioneer nature of the instructional videos development project, as well as the evaluation study, several limitations should be acknowledged. First, besides subjective outcome evaluation, the use of other evaluation strategies, such as objective outcome evaluation and qualitative evaluation, would produce useful findings [17]. For example, we can conduct a single group pre- and post-test study to evaluate students’ positive changes after watching the videos [18]. Moreover, collecting qualitative data is useful for us to further understand students’ subjective experiences of watching the videos. Various studies have used focus group or individual semi-structured interviews to examine students’ perceptions of newly developed teaching and learning activities and generated results that are conceptually and practically significant [67,68]. Second, as this is the first attempt to validate the instructional videos, replications of the findings at a different time using different samples are needed. As pointed out by Schmidt [69], replication is much needed in social science research to control for errors or to generalize research findings to a different or a larger population. Third, besides university students, teachers’ perceptions of the 24 videos’ value and effectiveness could offer us a new perspective to understand the value of videos in college-level leadership education. A recent study by Toto and Limone [70] also demonstrated the importance of teachers’ perceptions to the implementation of distance learning in college during the pandemic.

## 5. Conclusions

The present study demonstrates the use of instructional videos to educate college students on leadership knowledge based on the positive youth development approach. Strengthening young people’s positive youth development attributes could enhance their healthy development and cultivate their leadership qualities. A subjective outcome evaluation of these videos using a large-sample student survey was conducted. Two scales were developed to measure students’ perceptions of the content, implementation and effectiveness of the animated videos and case-based videos, respectively. Confirmatory factor analyses showed that the two scales possessed good statistical validity and reliability. The evaluation results suggested that the majority of students had highly positive perceptions of the videos’ content, delivery and the benefits gained from watching them. This study supports the use of instructional videos in university leadership education and offers insights on how colleges and universities could strengthen young people’s healthy development from a positive youth development perspective. As there is a need to conduct more studies on the quality of life (such as academic satisfaction) in Chinese people [71], the present study is a constructive response.

## Figures and Tables

**Table 1:** Description of the 15 Animated Videos.

Video Topic	Video Number	Description
Self-Leadership	A1 (*N* = 924)	Self-Understanding and Self-Management: The video introduces concepts and theories of self-leadership, including self-understanding and self-management. It also covers emotional management skills and steps to cope with negative emotions.
Cognitive Competence	A2 (*N* = 901)	Cognitive Competence: The video introduces concepts and examples related to misinformation, disinformation and propaganda. Students work with their peers to find daily examples of misinformation, disinformation or propaganda.
Social-Emotional Competence	A3 (*N* = 1039)	Social-Emotional Competence: This video explains the definition of social-emotional competence and the four-dimensional framework of social-emotional competence.
	A4 (*N* = 1012)	Egocentrism: This video presents theories related to egocentrism and illustrates the concept by analyzing the performance of an egocentric person.
Resilience	A5 (*N* = 1092)	Resilience and Resilient Qualities: This video introduces definitions of resilience and the concepts of internal and external resilient qualities that form protective factors of a person.
	A6 (*N* = 1076)	Richardson’s Model of Resilience: This video introduces Richardson’s resiliency model that highlights the process of resilience development.
Moral Competence	A7 (*N* = 1114)	Moral Development: This video illustrates Kohlberg’s Theory of Moral Development (including Heinz’s dilemma) and explains the three levels and six stages of moral development with real-life examples.
	A8 (*N* = 1105)	Is “Ghostwriting” Acceptable? This video describes the phenomenon of hiring ghostwriters among university students. It also introduces definitions and examples of ghostwriting and contract cheating.
Spirituality	A9 (*N* = 1069)	Spiritual Leadership Model: The video introduces the Spiritual Leadership Model and explains each component with the support of daily examples. This model explains why spiritual development is the fundamental need of both leaders and followers.
Law-Abiding Leadership	A10 (*N* = 971)	Concepts of Law-abiding Leadership: This video introduces the basic concepts and theories related to law-abiding leadership and socially responsible leadership.
	A11 (*N* = 968)	The Importance of Law-abiding Leadership: In this video, the importance of law-abiding leadership from the individual and organizational levels is discussed.
Cultural Competence	A12 (*N* = 1083)	Definitions and Theories of Cultural Competence: The video introduces definitions and models of cultural competence and encourages students to reflect on cross-cultural differences.
Effective Communication	A13 (*N* = 1025)	REPAIR Strategies: This video introduces the six steps in the REPAIR strategies for resolving interpersonal conflicts and invites student to share their opinions about the REPAIR strategies.
Team Building	A14 (*N* = 1046)	Five Conflict Resolution Strategies: This video introduces five conflict-handling modes in the Thomas-Kilmann Model of Conflict Management and discusses the effectiveness of these conflict-handling modes in different situations.
	A15 (*N* = 1040)	Win-Win Strategies: This video introduces the four steps to reach a win-win situation in the Conflict Resolution Model. In addition, the four typical win-win solutions are explained.

**Table 2:** Description of the Nine Case-based Videos.

Lecture Topic	Video Number	Description
Self-Leadership	C1 (*N* = 898)	Anger/Hatred Management Arising from Differences in Viewpoint: This role-play video presents a conflict between two good friends over their lifestyle choices. Students analyze emotional management in the case and share similar experiences of their own.
Cognitive Competence	C2 (*N* = 875)	Myths of MMR Vaccine: The video presents two distinct views towards the MMR vaccine (a vaccine against measles, mumps and rubella). Students analyze the arguments in the case and propose ways to distinguish true information from misinformation.
Social-Emotional Competence	C3 (*N* = 1007)	Story of a Family Issue: This role-play video uses storytelling technique to present a conflict between a mother and a daughter over the choice of television program at home. Students reflect on the concepts of social awareness and relationship management and discuss ways to handle the situation in the case.
Resilience	C4 (*N* = 1073)	A Story of Resilience (Eric Ma, a life-fighter): The video uses storytelling technique to describe the challenges faced by Eric Ma, who suffers from a rare disease that affects his normal functioning and appearance. Students identify the resilient qualities shown in the case and reflect on their own experiences of using coping strategies to handle changes and stressful events.
Spirituality	C5 (*N* = 1065)	Cases of Forgiveness: In this role-play video, three students with different experiences of betrayal and forgiveness are interviewed. Students reflect on their own experiences of forgiveness.
Cultural Competence	C6 (*N* = 1080)	How to Live Harmoniously with People from Different Cultural Background? This case-based video is an authentic interview with an ethnic minority family in Hong Kong on their difficulties. Students discuss issues encountered by people from different cultural backgrounds in Hong Kong.
Effective Communication	C7 (*N* = 1022)	Relationship Repair with Peers: The video presents a case about resolving conflict with peers with reference to face-enhancing strategies and face-detracting strategies. Students share their own or others’ experiences of using face-enhancing strategies to resolve conflict.
Team Building	C8 (*N* = 598)	Absence of Trust: This video presents problems in a dysfunctional team where team members are hesitant to discuss with each another. Students identify problems in the team and propose solutions based on related concepts and theories.
	C9 (*N* = 611)	Avoidance of Accountability: This video presents problems in a dysfunctional team with a free rider. Students identify problems in the team and propose solutions based on related concepts and theories.

**Table 3:** CFA findings of the 12-item SOES for the 15 Animated Videos.

Model	Description	χ^2^	df	CFI	NNFI	SRMR	RMSEA [90% CI]	AVE	CR	α
M1	L2 Self-understanding (*N* = 924)	352.10	54	0.94	0.92	0.04	0.08 [0.07, 0.09]	0.65	0.96	0.96
M2	L3 Cognitive competence (*N* = 901)	290.51	54	0.94	0.93	0.03	0.07 [0.06, 0.08]	0.69	0.96	0.96
M3	L4 Social-emotional (*N* = 1039)	301.66	54	0.95	0.94	0.03	0.07 [0.06, 0.07]	0.73	0.97	0.97
M4	L4 Introduction to egocentrism (*N* = 1012)	186.24	54	0.98	0.97	0.02	0.05 [0.04, 0.06]	0.76	0.97	0.98
M5	L5 Resilience (*N* = 1092)	245.38	54	0.96	0.95	0.03	0.06 [0.05, 0.06]	0.71	0.97	0.97
M6	L5 Richardson’s model (*N* = 1076)	170.82	54	0.98	0.97	0.02	0.05 [0.04, 0.05]	0.73	0.97	0.97
M7	L6 Moral development (*N* = 1114)	271.57	54	0.97	0.96	0.02	0.06 [0.05, 0.07]	0.77	0.98	0.98
M8	L6 Is ghostwriting acceptable (*N* = 1105)	118.20	54	0.99	0.99	0.01	0.03 [0.03, 0.04]	0.77	0.98	0.98
M9	L7 Spiritual leadership model (*N* = 1069)	155.61	54	0.98	0.98	0.01	0.04 [0.03, 0.05]	0.79	0.98	0.98
M10	L8 Concept of law-abiding (*N* = 971)	240.76	54	0.97	0.96	0.02	0.06 [0.05, 0.07]	0.86	0.99	0.99
M11	L8 Importance of law-abiding (*N* = 968)	198.27	54	0.98	0.97	0.01	0.05 [0.05, 0.06]	0.86	0.99	0.99
M12	L9 Cultural competence (*N* = 1083)	203.75	54	0.97	0.97	0.02	0.05 [0.04, 0.06]	0.81	0.98	0.98
M13	L10 REPAIR strategies (*N* = 1025)	156.60	54	0.98	0.98	0.01	0.04 [0.04, 0.05]	0.82	0.98	0.98
M14	L11 Conflict resolution (*N* = 1046)	229.47	54	0.97	0.97	0.02	0.06 [0.05, 0.06]	0.82	0.98	0.98
M15	L11 Win-win strategies (*N* = 1040)	162.94	54	0.98	0.98	0.01	0.04 [0.04, 0.05]	0.82	0.98	0.98

Note. α = Cronbach’s α (reliability). L = Lecture number.

**Table 4:** CFA findings of the 14-item SOES for the Nine Case-Based Videos.

Model	Description	χ^2^	df	CFI	NNFI	SRMR	RMSEA [90% CI]	AVE	CR	α
M16	L2 Anger management (*N* = 898)	418.16	77	0.93	0.92	0.04	0.07 [0.06, 0.08]	0.66	0.96	0.97
M17	L3 Myths of MMR vaccine (*N* = 875)	403.79	77	0.94	0.93	0.04	0.07 [0.06, 0.08]	0.68	0.97	0.97
M18	L4 Story of a family issue (*N* = 1007)	313.69	77	0.96	0.95	0.03	0.06 [0.05, 0.06]	0.74	0.98	0.98
M19	L5 Story of resilience (*N* = 1073)	520.15	77	0.93	0.92	0.04	0.07 [0.07, 0.08]	0.70	0.97	0.97
M20	L7 Case of forgiveness (*N* = 1065)	318.86	77	0.97	0.96	0.02	0.05 [0.05, 0.06]	0.78	0.98	0.98
M21	L9 Diverse cultural background (*N* = 1080)	342.83	77	0.96	0.95	0.02	0.06 [0.05, 0.06]	0.80	0.98	0.98
M22	L10 Relationship with peers (*N* = 1022)	232.78	77	0.98	0.97	0.02	0.04 [0.04, 0.05]	0.81	0.98	0.98
M23	L11 Absence of trust (*N* = 598)	264.36	77	0.95	0.94	0.02	0.06 [0.06, 0.07]	0.82	0.98	0.99
M24	L11 Avoidance of accountability (*N* = 611)	258.28	77	0.96	0.95	0.02	0.06 [0.05, 0.07]	0.81	0.98	0.98

Notes. α = Cronbach’s α (reliability). As there are two case-based videos for Lecture 11, half of the students were required to watch and evaluate “Absence of trust”, while the other half were required to watch and evaluate “Avoidance of accountability”. L = lecture number.

**Table 5:** Positive Responses Rates of Post-Lecture Video Evaluation for the 15 Animated Videos on Theories/Concepts.

Questionnaire Item		A1 *N* = 924	A2 *N* = 901	A3 *N* = 1039	A4 *N* = 1012	A5 *N* = 1092	A6 *N* = 1076	A7 *N* = 1114	A8 *N* = 1105	A9 *N* = 1069	A10 *N* = 971	A11 *N* = 968	A12 *N* = 1083	A13 *N* = 1025	A14 *N* = 1046	A15 *N* = 1040
		Positive Response Rate %	Positive Response Rate %	Positive Response Rate %	Positive Response Rate %	Positive Response Rate %	Positive Response Rate %	Positive Response Rate %	Positive Response Rate %	Positive Response Rate %	Positive Response Rate %	Positive Response Rate %	Positive Response Rate %	Positive Response Rate %	Positive Response Rate %	Positive Response Rate %
1	The animated video is well designed.	95.95	98.20	96.69	98.02	97.96	97.39	98.19	97.73	96.86	94.78	94.69	96.82	97.14	97.58	97.87
2	The animated video is relevant to the lecture topic.	98.58	98.55	98.45	98.91	98.62	98.88	98.20	98.01	97.83	94.62	95.74	97.97	97.95	97.88	97.78
3	The animated video is easy to understand.	97.60	98.43	98.15	98.51	98.25	97.11	97.74	98.09	96.06	95.43	95.12	96.94	97.75	98.18	98.26
4	The animated video is interesting.	90.81	94.19	93.02	95.43	95.48	95.26	95.78	96.65	94.00	92.13	91.41	94.89	96.09	95.59	95.75
5	The teacher used the animated video effectively.	96.61	97.65	97.86	98.01	98.26	98.60	97.74	98.28	97.46	95.03	95.12	97.59	97.25	97.70	97.87
6	The animated video has enabled me to understand related concepts.	97.28	98.55	98.45	97.72	98.71	98.23	98.56	97.82	96.44	95.03	94.91	98.05	97.56	98.17	98.08
7	The animated video has deepened my understanding of the topic.	97.71	98.20	97.68	97.82	98.43	98.42	98.00	98.27	97.09	94.42	94.82	97.59	97.46	97.12	98.27
8	The animated video has enhanced my interests in the topic.	90.62	94.20	94.10	95.33	94.86	95.24	95.75	96.64	94.28	90.88	91.39	94.99	95.79	94.26	96.62
9	The animated video has helped me to reflect on the topic.	95.41	96.31	96.99	96.92	96.77	97.75	97.11	97.45	96.70	92.46	93.34	96.29	97.45	97.40	97.69
10	I prefer more such animated video in the teaching and learning of this subject.	95.39	95.76	96.23	96.82	97.13	96.82	97.57	97.46	96.90	94.11	94.72	96.48	95.99	96.82	97.49
11	The animated video can benefit my development.	95.71	96.85	96.52	97.80	97.50	97.76	97.84	96.99	96.61	93.70	93.58	97.13	97.94	97.41	97.40
12	Overall speaking, I am satisfied with the animated video.	97.48	98.19	98.06	98.31	98.52	98.22	98.29	98.09	96.91	94.42	94.49	97.67	97.65	97.69	97.68

Notes. (items 1 to 12) All items were rated on a 6-point Likert scale with 1 = Strongly Disagree, 2 = Disagree, 3 = Slightly Disagree, 4 = Slightly Agree, 5 = Agree, 6 = Strongly Agree.

**Table 6:** Positive Responses Rates of Post-Lecture Video Evaluation for the Nine Case-based Videos (Application and Discussion).

Questionnaire Item		C1 (*N* = 898)	C2 (*N* = 875)	C3 (*N* = 1007)	C4 (*N* = 1073)	C5 (*N* = 1065)	C6 (*N* = 1080)	C7 (*N* = 1022)	C8 (*N* = 598)	C9 (*N* = 611)
		Positive Response Rate %	Positive Response Rate %	Positive Response Rate %	Positive Response Rate %	Positive Response Rate %	Positive Response Rate %	Positive Response Rate %	Positive Response Rate %	Positive Response Rate %
1	The video-based activity is well designed.	95.09	97.82	96.42	97.75	96.23	97.68	97.14	97.31	98.04
2	The video-based activity is relevant to the topic.	98.55	98.97	98.31	98.60	97.74	98.24	98.03	98.32	98.69
3	The video is easy to understand.	97.32	98.62	97.41	98.31	97.17	97.59	98.23	98.49	98.19
4	The content of the video is interesting.	92.48	96.21	94.29	96.44	95.31	96.46	95.28	95.64	97.02
5	The content of the video is stimulating.	96.20	97.93	96.41	97.48	96.05	97.02	96.76	97.48	98.02
6	The video-based activity has increased my interaction with the teacher.	86.32	90.39	91.62	91.50	93.50	93.76	94.28	94.47	96.39
7	The video-based activity has increased my interaction with classmates.	82.60	89.43	92.12	89.79	91.89	93.11	93.71	93.63	94.74
8	The teacher has used the video effectively.	96.98	98.05	97.99	98.03	97.55	98.04	97.44	97.99	98.52
9	I have been very engaged in the video-based activity.	92.61	94.10	94.49	95.97	95.19	96.56	95.39	96.65	97.87
10	The video-based activity has deepened my understanding of the topic.	96.87	98.28	97.60	98.32	97.26	97.49	97.44	98.32	98.03
11	The video-based activity has enabled me to apply the theories in real life cases.	93.60	96.89	96.49	96.06	96.41	97.67	96.37	96.98	97.54
12	The video-based activity has enhanced my interests in the topic.	92.84	95.52	95.30	96.26	95.56	95.73	96.18	95.64	97.21
13	The video has helped me reflect on the subject matters.	96.07	97.11	97.20	98.04	96.61	97.59	97.35	97.99	97.54
14	I prefer more such video-based activities in the teaching and learning of this subject.	94.08	97.13	96.90	97.75	96.89	96.83	96.76	96.96	97.21

Notes. (items 1 to 14) All items were rated on a 6-point Likert scale with 1 = Strongly Disagree, 2 = Disagree, 3 = Slightly Disagree, 4 = Slightly Agree, 5 = Agree, 6 = Strongly Agree.

**Table 7:** Positive Responses Rates of Post-Lecture Video Evaluation for Lectures 2 to 11 (Two Items Evaluating the Effectiveness of the Lecture).

Lecture Topic		Self-Leadership	Cognitive Competence	Socio-Emotional Competence	Resilience	Moral Competence	Spirituality	Law-Abiding Leadership	Cultural Competence	Effective Communication	Team Building
Video Number		A1 and C1 (*N* = 892)	A2 and C2 (*N* = 867)	A3, A4 and C3 (*N* = 999)	A5, A6 and C4 (*N* = 1072)	A7 and A8 (*N* = 1105)	A9 and C5 (*N* = 1062)	A10 and A11 (*N* = 964)	A12 and C6 (*N* = 1076)	A13 and C7 (*N* = 1019)	A14, A15, C8 and C9 (*N* = 1040)
Questionnaire Item		Positive Response Rate %	Positive Response Rate %	Positive Response Rate %	Positive Response Rate %	Positive Response Rate %	Positive Response Rate %	Positive Response Rate %	Positive Response Rate %	Positive Response Rate %	Positive Response Rate %
1	Overall speaking, the lecture has enriched my development.	96.61	97.44	98.18	98.78	97.63	97.62	92.77	97.56	98.11	97.39
2	On the whole, I am very satisfied with this lecture.	96.26	98.15	97.88	97.65	97.82	97.26	92.71	97.58	98.04	97.68

Notes. (items 1 to 2) All items were rated on a 6-point Likert scale with 1 = Strongly Disagree, 2 = Disagree, 3 = Slightly Disagree, 4 = Slightly Agree, 5 = Agree, 6 = Strongly Agree.

## Data Availability

The data presented in this study are available on request from the corresponding author.
